# The Importance of Human Emotions for Wildlife Conservation

**DOI:** 10.3389/fpsyg.2020.01277

**Published:** 2020-06-24

**Authors:** Nathalia M. Castillo-Huitrón, Eduardo J. Naranjo, Dídac Santos-Fita, Erin Estrada-Lugo

**Affiliations:** ^1^El Colegio de la Frontera Sur – Unidad San Cristóbal, San Cristóbal de Las Casas, Mexico; ^2^Instituto Amazônico de Agriculturas Familiares, Universidade Federal do Pará, Belém, Brazil

**Keywords:** anger, disgust, fear, happiness, mammals, reptiles

## Abstract

Animals have always been important for human life due to the ecological, cultural, and economic functions that they represent. This has allowed building several kinds of relationships that have promoted different emotions in human societies. The objective of this review was to identify the main emotions that humans show toward wildlife species and the impact of such emotions on animal population management. We reviewed academic databases to identify previous studies on this topic worldwide. An analysis of the emotions on wildlife and factors causing them is described in this study. We identified a controversy about these emotions. Large predators such as wolves, coyotes, bears, big felids, and reptiles, such as snakes and geckos, promote mainly anger, fear, and disgust. This is likely due to the perceptions, beliefs, and experiences that societies have historically built around them. However, in some social groups these animals have promoted emotions such as happiness due to their values for people. Likewise, sadness is an emotion expressed for the threatening situations that animals are currently facing. Furthermore, we associated the conservation status of wildlife species identified in the study with human emotions to discuss their relevance for emerging conservation strategies, particularly focused on endangered species promoting ambiguous emotions in different social groups.

## Introduction

Since our origins, wildlife has always had a very important role in human life. The very diverse and continuous human–wildlife interactions can be seen from three main perspectives: (1) Utilitarian, in which wild species provide goods for human well-being, such as food, clothing, transport, tools, raw materials, and companionship, among others; (2) Affective, where human beings feel sympathy, admiration, and respect for animals because of religious, mystical, or philosophical reasons (Kellert et al., 1996), which has greatly contributed to cultural development worldwide (Herzog and Galvin, 1992; Alves, 2012); and (3) Conflictive, because of the real or potential damage that wild species may inflict on people and their interests (e.g., attacks on humans, livestock predation, damage on crops, and infrastructure, among others; Lescureux and Linnell, 2010). Human–wildlife conflicts have motivated animal killings for centuries, which in many cases continue nowadays (Woodroffe, 2001).

Human–wildlife relationships have relied on the uses, values, and meanings that animals represent for people through time and space in different cultures (Driscoll, 1995; Prokop et al., 2010). Societies have developed a cultural predisposition for emotional reactions toward wild animals (Kellert and Wilson, 1993), causing either positive or negative effects depending on the species (York and Longo, 2017). Fear, anger, and disgust are emotions generating attitudes and behaviors against the presence of some species (Fritts et al., 2003; Jacobs, 2012). In contrast, emotions, such as happiness, which comes out when cherished species are seen in a given place, or sadness before the vulnerability of others, may generate positive attitudes for their conservation (Prinz, 2004). This relationship between human emotions and attitudes has an effect on the presence, absence, and recovery of wildlife populations (Herzog and Burghardt, 1988). Understanding the transcendence of the emotional factors triggered by animals on human beings would improve our knowledge on the human dimensions of wildlife conservation. In this paper, we offer an overview of the influence that emotions have had on the relationships between wildlife and people through time. A substantial amount of the literature reviewed consists of studies conducted on large carnivores in Europe, such as the brown bear (*Ursus arctos*) and the wolf (*Canis lupus*), as well as on snakes around the world. We analyze and discuss relevant aspects that could be considered in further studies on threatened and culturally relevant animal species across Latin American countries.

## Origins of Emotions Toward Wildlife

Darwin (1897) recognized that emotions are manifested by all persons throughout their lifetime, but they vary in an individual between different moments of its life span. Frijda and Mesquita (1998) mentioned the main points characterizing the emotions in their theoretical perspective: (1) emotions are considered individual responses to relevant events producing feelings of pleasure or pain; (2) they help to find solutions to concerns that cannot be treated routinely; (3) they are always about something, they are used to accept or decline the interaction with a real or imagined object, person, or wild animal in this case; (4) they tend to control behaviors and thoughts (e.g., angry impulses, behaviors, and thoughts); and (5) emotions are correlated with psychological, physiological, and social components establishing, changing, or maintaining a particular relationship with a specific object in a concrete situation.

There is a wide array of studies analyzing human emotions, their origins, functions, and presence in human life (Ekman, 1999; Plutchik, 2001a; Nummenmaa et al., 2014, among many others). Six basic emotions have been proposed: happiness, surprise, disgust, anger, fear, and sadness (Ekman et al., 1969). Izard (2009) suggested classifying emotions into two groups: “positive,” representing interest and joy (happiness and surprise), and “negative,” including anger, disgust, fear, and sadness. This classification is an artifact of traditional psychology not informed about an evolutionary approach. Here, we have focused on basic emotions to explain human–wildlife relationships because secondary emotions (the combination of basic emotions) are more useful for assessing social relationships among human beings (Harelli and Parkinson, 2008).

In this review, we consider two different approaches to explain the origins of basic emotions aiming to understand human–wildlife relationships through time. The first is the evolutionary approach, which suggests that emotions have evolved to solve adaptive problems in different environments (Plutchik, 2001b), such as social communication, reproduction processes, and mechanisms for information processing leading to behavioral responses to specific events or objects (Al-Shawaf et al., 2016). Predator presence could have been one of such events contributing to the evolution of human emotions and the development of physiological, psychological, and morphological responses for survival (Öhman and Mineka, 2001; Prokop and Randler, 2018). In particular, fear and disgust are adaptive emotions helping to react toward something representing a risk for human life (Ekman and Cordaro, 2011). Fear and disgust, for instance, have been the most studied emotions due to their implications for human survival since the origin of our species (Polák et al., 2019). Fear probably was a defense mechanism against dangerous animals, particularly large predators (Öhman, 1986; Dalgleish, 2004). It is believed that potential alert signals emitted by human groups facing predators, with whom they coexisted and sometimes competed for space, water, prey, and other resources, triggered physiological reactions such as heart rate increase, profuse sweating, and pupil dilation, allowing the generation of alert responses. In that way, human beings have historically developed greater awareness toward potentially perilous animals, such as snakes and spiders (Öhman et al., 2001; Öhman and Mineka, 2003; LoBue and DeLoache, 2008). This adaptation mediated by fear has probably been genetically fixed throughout generations, provoking the innate physiological responses mentioned above when dangerous species are or could be present (Öhman, 1986). The amygdala is the brain region where fear-generating stimuli are processed into a strong reaction that in some cases may affect human vision (Phelps et al., 2006). On the other side, disgust can help protect the individual against infections and disease (Curtis et al., 2011). Disgust is saved in memory to avoid future exposure to the subject, in this case with potentially threatening animals (Al-Shawaf et al., 2016).

The second approach explaining the origins of basic emotions is the cultural context, where people integrate their physical environment with individual and collective experiences, perceptions, meanings, attitudes, and animal-related traditions to construct emotional diversification (Prinz, 2004; Johansson et al., 2012). In this view, it can be said that human emotions associated with wildlife have evolved over time and continue to be gradually built and rooted in our societies all over the world. Under the cultural context approach, emotions can be understood on two levels: (1) the individual level, involving meanings, beliefs, attitudes, and behaviors based on personal experiences, knowledge, and perceptions, and (2) the social level, where emotions are determined by collective factors such as experiences, meanings, beliefs, and myths typical of a certain region or culture, which are transmitted among individuals throughout generations (Ekman, 1999; Prinz, 2004).

## Species-Specific Emotions

Physical characteristics of wildlife species and their “personalities” created by humans have generated a variety of emotions (Kellert et al., 1996; Kruuk, 2002; Prokop and Randler, 2018). Emotions such as fear and anger may be induced by predators that are bigger and heavier than persons, as in the case of large carnivores (e.g., bears, wolves, and big cats) (Røskaft et al., 2003) or by those species unattractive for most people, like worms, small carnivores, bats, and reptiles, which are often perceived as harmful (Knight, 2008; Prokop and Tunnicliffe, 2008; Prokop et al., 2009). In contrast, beloved animals such as colorful birds or small herbivore mammals (e.g., rabbits) may cause happiness providing they are not noxious for people or their livelihoods (Prokop and Kubiatko, 2008). However, these animals are sometimes perceived in different ways. For some social groups (e.g., farmers), small mammals such as rabbits as rodents may represent a threat due the damage they can inflict on crops, cattle, properties, and human health (Morzillo and Merting, 2011; Breed and Moore, 2016). Actual or potential damage can promote negative attitudes motivated by emotions of anger, disgust, and fear.

Animal body shape is another physical feature that has been found to be important for the expression of emotions such as fear and disgust. In the case of class Reptilia, two groups could be recognized by people according with their similar morphotype (with legs and legless). Reptiles with legs (lizard, turtle, and crocodile) tend to cause fear in many people; crocodiles, specially generate intense fear in many people, in part because of the number of attacks occurring worldwide (CrocBITE, 2020). In contrast, legless reptiles (e.g., amphisbaenia and *Larutia*) that have thin bodies, smooth textures, small eyes, and dull colorations generate disgust (Janovcová et al., 2019; Rádlová et al., 2019). Specifically, snakes have long bodies, scales with contrasting patterns, bright coloration, and silent, rolling movements that immediately calls up human attention (LoBue and DeLoache, 2008, 2011; Rádlová et al., 2019). It is likely that both fear and disgust can be simultaneously felt by a person observing a particular species (Rádlová et al., 2019). The ample diversity of snakes around the world makes it difficult to generalize emotions across cultures toward different taxa.

Species coloration has been an attribute to help identify dangerous animals (Prokop and Fančovičová, 2013), allowing emotional responses in human beings (Öhman, 1986). Striking color (“aposematic”) combinations such as bright red and black in some snakes and spiders intensify fearful reactions (Öhman and Mineka, 2003; LoBue and DeLoache, 2011; Prokop et al., 2018). On the other hand, it has been reported that striking coloration allowed perceiving snakes as beautiful animals (Marešová et al., 2009) in spite they are fearsome (Janovcová et al., 2019). It is noteworthy that aposematic species are simultaneously fearsome and attractive particularly for young persons between 10 and 20 years of age, promoting their interest in those animals (Prokop and Fančovičová, 2013). On the other hand, animals’ coloration could be attractive for humans and motivate “positive” feelings. In this sense, Lišková et al. (2015) discovered that hues of blue and green in birds of the Pittidae family promote human preference. Psychologists have found that green is usually associated with happiness, relaxation, and comfort because it is related to nature, while blue elicit happiness, relaxation, and peacefulness, among other feelings (Kaya and Epps, 2004). However, human affection for birds also represents a pressure for wild populations, especially for those charismatic species used as pets, promoting illegal trade (Alves et al., 2013).

Feeding habits of species may also influence emotions: large predators are usually regarded as hazardous and fearsome, while their prey provoke sadness (Prokop and Kubiatko, 2008). Large herbivores and omnivores in some places are often seen as less fearsome than strict carnivores. This is the case of the mainly vegetarian brown bear (*Ursus arctos*) in some regions of Europe (Lescureux and Linnell, 2010). However, in other areas and cultures, large herbivores such as elephants (*Loxodonta africana*) cause intense emotions of anger and fear because of the damage they inflict on crops and rural villages (Lamarque et al., 2009). Although “dangerous” animals promote the attention of people (Prokop and Randler, 2018), it is interesting to note that human emotions may vary depending on the life stage of the animal. For example, jaguar (*Panthera onca*) cubs and lion (*Panthera leo*) cubs are perceived as lovely and safe animals given their physical features, causing minor concern in societies, while adult jaguars and lions are generally considered less attractive and very dangerous, promoting fear (Knight, 2008). This trend is also reported for amphibians, for which people show more disgust toward the adult stage than for tadpoles (Prokop and Fančovičová, 2012).

Venom in animal species is one of the most remarkable features triggering fear across cultural groups. As a consequence, snakes constitute an interesting case study in which most species produce fear all over the world, although particular species are in fact perceived as beneficial due to their role as controllers of agricultural pests, producing positive feelings in local farmers (Ballouard et al., 2013). In this regard, Ballouard et al. (2013) observed different intensities of fear toward selected snake groups (cobras, vipers, and boas) depending on the nationality and cultural background of their interviewees.

Animal activity patterns constitute one more physical factor influencing human emotions toward wildlife. Humans are not adapted for living in the darkness; they have a poor vision to act in this kind of environment, hence they may associate nocturnal species such as felines, some snakes, rodents, and bats with danger (Buss, 2016). In addition, these animals historically have been linked to “evil forces” damaging human beings worldwide (Prokop et al., 2009). Contrastingly, many diurnal species (e.g., most of the birds and ungulates) are usually related to positive values such as peacefulness and wisdom that have inspired leaders and rulers to make better decisions (Cano-Contreras, 2009).

Physical characteristics have been useful to classify animals depending on the emotions they produce on people. In this sense, tarantulas, snakes, sharks, and mosquitoes have been categorized as perilous, generating agonistic emotions. Contrastingly, large, charismatic species that have traditionally been regarded as dangerous but intelligent at the same time motivate emotions that may result in actions for their protection, as it has occurred for lions (*Panthera leo*), tigers (*Panthera tigris*), leopards (*Panthera pardus*), and polar bears (*Ursus maritimus*) (Driscoll, 1995; Landová et al., 2018). These categories have emerged after the anthropomorphization of animals, a process in which cultural groups attribute human features and “personalities” to wildlife species (Kruuk, 2002). For instance, the panda bear (*Ailuropoda melanoleuca*) inspires tenderness and happiness when it is observed, but those emotions are overcome by sadness after considering its high vulnerability to extinction. In this case, positive attributes facilitate particular species to become flagships for wildlife conservation (Root-Bernstein et al., 2013).

In rural communities where people frequently interact with wildlife, knowledge about the behavior of culturally relevant species develops better than in other areas. This facilitates the anthropomorphization of certain animals calling them “shy,” “noxious,” and “monstrous,” among other adjectives, which intensifies fear and rejection toward them (Lescureux and Linnell, 2010). Furthermore, if the presence of an animal implies economic losses for residents of a community, their predominant perception will be negative and will produce anger that may end in lethal management (Naughton-Treves, 1997). Contrastingly, animals inspiring greatness and qualified as “kings” of the wilderness will likely motivate local people to feel happiness and pride because of their presence in the region (Lescureux and Linnell, 2010). These examples help identifying the relevance of animal physical features in emotions, which transform throughout history according to the natural, social, and economic context of each human generation. In some cases, emotions produce attitudes against the conservation of unpopular species (Knight, 2008). Therefore, we propose to highlight the ecological role of dangerous or disgusting species as a potential way to mitigate negative emotions toward them.

## Emotions and Sociodemography

Emotions induced by wildlife differ among individuals according to variables such as their sex, age, cultural and natural environment, and perceived vulnerability to each species (Johansson et al., 2012). It has been shown that young children (under 3 years of age) of both sexes take more time to detect a snake and react toward it than their parents (LoBue and DeLoache, 2011). That behavior was explained by DeLoache and LoBue (2009), proposing that fear and alert signals in front of this kind of animals develop later, when individuals start to explore their environment and link adult behaviors with animal species.

Fear and disgust have been the most studied emotions between genders. In general, women tend to express stronger negative emotions (fear and disgust) toward invertebrates, amphibians, predatory mammals like bears, wolves, lynx (*Lynx lynx*), and wolverine (*Gulo gulo*) and toward snakes compared to men (Öhman and Mineka, 2003; Røskaft et al., 2003; Ballouard et al., 2013; Bajwa et al., 2014; Prokop and Fančovičová, 2016; Prokop et al., 2016). This difference seems to be related to the female gender role taken since the start of human evolutionary history, where men developed skills for both hunting and escaping from predators (Prokop and Fančovičová, 2010). Likewise, men gradually reduced their fear of large animals, while women kept distance from those species in part because of their household activities and their care for children in safer places (Røskaft et al., 2003; Prokop et al., 2011). However, differences within genders are usually present in different cultural and geographic contexts (Kellert and Berry, 1987; Bjerke et al., 2001; de Pinho et al., 2014). In some societies, women, particularly adolescents, have a greater disposition to spend more time in wildlife related activities as compared to men (e.g., volunteer programs; Kidd and Kidd, 1997). This information could be useful to direct conservation programs in spaces as zoos where experiences with uncharismatic and endanger animals could help to promote positive emotions and attitudes.

Age is a significant variable determining the presence and intensity of agonistic emotions toward animals, which may be related to personal experiences. Childhood is the critical life stage when fear of predators starts and when attitudes and behaviors to avoid encounters with them develop (Öhman, 1986). It is likely that fear of predators intensifies with learning from parents, given that as the child gets older, his/her reactions become faster when facing species such as snakes (LoBue and DeLoache, 2008). In this regard, fear of animals may either decrease (Kaltenborn et al., 2006) or increase (Røskaft et al., 2003) with age.

Besides age, the natural and cultural environments in which an individual grows determine the knowledge, perceptions, and emotions related to animals (Frynta et al., 2011). For a person raised in close contact with nature, an encounter with a wild animal can induce happiness, while the same species may produce fear in an individual that has always lived far away from natural spaces (Kellert, 1993; Manfredo, 2008; Almarcha, 2019). The presence or absence of different species in human territories has a role in the generation of emotions. Residents of rural areas who frequently interact with wildlife are usually less fearful of animals than city dwellers. This is because closeness with native animals promotes knowledge about their ecology and behavior, allowing for building better management strategies and reactions toward them (Røskaft et al., 2003).

Likewise, recreational activities involving contact with wildlife such as hiking, bird watching, fishing, and hunting have direct influence in emotions, facilitating the overcoming of fears and phobias by promoting learning through first-hand experiences, although in some cases, these activities decrease with age (Bjerke et al., 2001; Røskaft et al., 2003; Prokop et al., 2011). In particular, emotions produced by hunting deserve further discussion. Subsistence hunting as a traditional practice in many rural areas of the world usually involves local regulations to avoid overexploitation and feelings of respect by the hunters toward their prey (e.g., Santos-Fita et al., 2015). In contrast, sport hunting is more focused on the pleasure of the hunter for finding and killing his target species, which has been a motive social dispute in different contexts, generating anger in broad sectors of society considering this an unacceptable practice (Nelson et al., 2016). Some of these recreational activities involve parents and their children, who get used to those practices at an early age (Amiot and Bastian, 2015). This can be an important inter-generational strategy to avoid negative attitudes toward fearsome and disgusting animals and promote positive emotions (i.e., happiness and surprise), especially in areas where human–wildlife conflicts may arise.

Significant differences have been found among people with different levels of study with respect to fear of wildlife species: individuals with higher levels of education are generally less fearful of wild animals than those with lower degrees of studies (Røskaft et al., 2003). It is likely that individuals with higher education had more opportunities to receive information on the environment and wild animals in particular, which may have reduced their negative prejudices and perceptions about non-charismatic species, maximizing their perspectives on the ecological benefits provided by those animals.

## Emotions Through Time and Space

The geographic space where an event occurs triggers distinct emotions, which have varied according to the lifestyles of societies (Mesquita and Frijda, 1992). This argument could be used to understand emotions historically induced by wildlife, considering the different worldviews of each culture. For example, snakes were regarded as deities in Mesoamerican cultures, including *Quetzalcoatl* or *Kukulkan* (the feathered serpent), which was the most important deity for the Aztecs and the Maya, respectively (Díaz, 2007). Snakes were also given high rankings among the deities of the ancient Greek, Egyptian, Hindu, and Roman civilizations, where some of these reptiles were associated with values of wisdom, justice, and power (Stanley, 2008; Al-Rawi, 2012). These reptiles have also starred countless stories and myths around the world (Ménez, 2003), but for Christians, Muslims, and Jews, snakes have traditionally represented evil and death (González, 2003; Al-Rawi, 2012). Nowadays, myths about the damage caused by snakes are important elements to promote and intensify fear in rural communities (Fita et al., 2010). The social fear could be learned, inherited, and used by societies across generations, driving particular attitudes toward wild species (Öhman, 1986). In this case, the relevant ecological role of snakes as predators and pest controllers has been largely neglected.

Another interesting example is that of wolves, which have been protagonists of many stories and myths worldwide. These carnivores have traditionally been portrayed as fearsome and dangerous animals, producing social rejection in most areas where they are present, nonetheless, in particular cases such as that of ancient Rome (whose founders were suckled by a she-wolf) and that of native North American cultures, for whom wolves were spiritual symbols related to power and intelligence (Fritts et al., 2003; Prokop et al., 2011).

Beyond mythology, other elements that have facilitated the development of cultures (e.g., art, literature, symbolism, religion) have had their foundations in the relationships between humans and wildlife, involving emotions promoting respect and admiration (Fritts et al., 2003; Alves, 2012; Almarcha, 2019). These emotions frequently lead to attitudes favorable for animal care and conservation.

Other events that have always happened, but which have received special attention in recent decades because of the human population growth and expansion, are the attacks of large carnivores on people and livestock, and crop damage by large herbivores (Inskip and Zimmermann, 2009). These events make jaguars, tigers, lions, leopards (*Panthera pardus*), hyenas (*Crocuta crocuta*), African wild dogs (*Lycaon pictus*), and African elephants (*Loxodonta africana*), among others, be considered problems in rural communities, giving place to misunderstandings and false beliefs about their behavior (Marchini and Macdonald, 2012; Dickman et al., 2014). This situation has contributed to magnification of the actual damages of those species, stimulating even more fear, disgust, and rejection toward them (Lescureux and Linnell, 2010).

In this sense, the individual background and experiences of humans contribute to their emotions and behaviors. For example, the presence of large predators may produce fear and thoughts of escape in most people, while some others may feel encouraged to confront the danger (Al-Shawaf et al., 2016). The context of the encounter with an animal may also be relevant for the emotions manifested. For a given person, the sighting of a carnivore such as a female puma with their offspring while hiking on a forest trail may produce fear and desire to escape. In contrast, the same person may feel surprised and delighted to have the same sighting from the safety of a car (narratives collected by the first author in Chiapas, Mexico). Furthermore, local knowledge and the emotional links between people and wildlife could be useful to identify flagship species to foster interest in nature (Bowen-Jones and Entwistle, 2002). Flagship species [e.g., giraffe (*Giraffa camelopardalis*), elephants, and lions, among others] are usually charismatic and popular and may be relevant for promoting positive emotions in a public that has been distant from wild animals. Differently, more complex sets of emotions (both positive and negative) are usually present where people are in constant interaction with these animal species (Bowen-Jones and Entwistle, 2002; de Pinho et al., 2014).

Zoos represent spaces where emotional confrontations take place. For instance, Marseille et al. (2012) observed visitors watching imposing and charismatic polar bears. The authors found that visitors felt happy in front of the bears, but at the same time they felt sad after recognizing the small size of the enclosures and the stereotyped behavior of the captive animals. Interestingly, visitors’ emotions transformed into fear and even greater sadness when they were told about observing polar bears in their natural habitat, which was associated with concerns about human safety and habitat vulnerability. Another element that has an effect in the affection of children for wild animals is the presence of pets (Bjerke et al., 2001). Pets can boost appreciation emotions, such as happiness, while naturalistic, ecological, humanistic, and moralistic attitudes may also be encouraged (Prokop and Tunnicliffe, 2010).

## Misinformation Causes a Mix of Emotions

Although knowledge about animals usually differs between urban and rural communities, the lack of accurate information about the species and their contribution to ecosystem services is persistent in both environments (Gomes et al., 2017). It promotes the intensification of emotions such as danger and disgust, especially for species that are unattractive to people. Disgust has also been identified as one of the emotions inducing human rejection. It may arise when people perceive nasty odors in animals, or when unpleasant feelings emerge while touching (or thinking about) the fur of certain mammals (Johansson et al., 2012) or the skins of amphibians such as frogs (LoBue and DeLoache, 2011). In other cases, disgust may be brought after linking animals such as spiders and rats with dirtiness, pollution, disease spreading, and potential crop damage (Kellert, 1993; Davey, 1994; Prokop and Tunnicliffe, 2010). Furthermore, animals that cause disgust are often perceived as ugly (Janovcová et al., 2019).

Contempt of human societies for amphibians and reptiles intensifies misinformation about them and favors negative attitudes toward them (Manzano-García and Martínez, 2017). For example, it has been documented that non-venomous snakes are killed just because of their resemblance to poisonous species (Breed and Moore, 2016). Moreover, misinformation is an intensifier of disgust, for instance, when considering geckos (*Hemidactylus turcicus*) as venomous animals or vectors of skin diseases (Ceríaco et al., 2011), or bats as a threat for fruit crops and responsible to infect people with parasites and viruses (Musila et al., 2018). In this sense, the case of bats and pangolins (Pholidota) could be cited, which are considered the main transmitting agents of the novel coronavirus (COVID-19; van Staden, 2020). The respiratory illness has become a pandemic infecting million and killing many thousands of people around the world (Nature, 2020). It is likely that the disease has a zoonotic origin as a result to the food and medicinal uses of animals (van Staden, 2020). Therefore, in some places there has been motivation to eliminate these animals (Zhao, 2020). This event might increase the negative perception and emotions of anger, disgust, and fear for this kind of animals and will encourage the eradication of populations without considering their importance in ecosystems. In this regard, it has been found that women and residents living near caves tend to believe in myths about bats more than men and people living far from caves (Musila et al., 2018).

## Biophilia Versus Biophobia

Fearsome and disgusting species frequently induce rejection attitudes in social groups (Öhman and Mineka, 2001), a phenomenon known as “biophobia” that is used to express the feeling of panic, fear, and disgust in front of a particular non-human living being. Phobia for animals (agrizoophobia) is one of the most frequently reported biophobias in the general population (Antony and McCabe, 2005), but there are actually around twenty-five documented phobias to particular animal groups, such as that for snakes (ophidiophobia), spiders (arachnophobia), insects (entomophobia or insectophobia), ants (myrmecophobia), bees (apiphobia or melissophobia), and birds (ornithophobia), among others (Fredrikson et al., 1996; Antony and McCabe, 2005; Prokop and Fančovičová, 2013). However, there are no specific phobias for carnivores, probably because the coevolution between humans and these animals has been too short in comparison with other groups such as snakes (Prokop and Randler, 2018).

Biophobia may promote persecution and extermination attitudes (Zhang et al., 2014). Avoiding contact with animals or killing them are the most frequent reactions without considering their long-term impacts on ecosystems (Antony and McCabe, 2005; Al-Shawaf et al., 2016). Orr (1993) mentioned that one of the causes of biophobia is social distancing from nature. In a parallel way, biophilia has a genetic basis and consists of the interest and empathy of humans for other living beings (Wilson, 1993). As industrialization and urbanization increase around the world, lifestyles change in human societies, sometimes in radical ways (Steffen et al., 2008). These processes have contributed to the distancing of people from their natural environment even in rural communities (Louv, 2008; Lescureux and Linnell, 2010). However, there are still spaces such as zoos and natural parks facilitating social approach and understanding of wildlife in most of the cities and large towns all over the world. In those spaces, visitors are generally safe in front of animals that otherwise would be considered dangerous or harmful, and they may feel sadness and even culpability after recognizing the impact of the human population on those species. In this sense, Vining (2003) suggested that visiting zoos and natural parks may represent opportunities for reconnecting people and wildlife to enhance social cooperation in conserving biodiversity.

## Emotions and Wildlife Conservation

Human emotions transcend over time. A specific emotion is saved by the individual as an experience that may be used in future behavior and decision-making (Izard, 2009). Protection attitudes toward spiders, insects, amphibians, and reptiles are milder than those shown for other groups, such as birds and mammals due the sentiments of danger or disgust that these animal groups provoke in humans (Prokop and Fančovičová, 2013; Prokop et al., 2016). In addition, emotional experiences may have an effect on wildlife management techniques (Larson et al., 2015). This has occurred during experiences of invasive species management. One example is that of the house sparrow (*Passer domesticus*), which competes for food and space with native birds and generates anger or disgust when managed through nest and egg removal, repellents, and traps. In contrast, bluebirds (*Sialia sialis*) stimulate happiness in people watching them and listening to their songs, who at the same time feel sadness for these birds due to the negative impact of human activity on their populations. These feelings motivate protection attitudes favoring the persistence of the liking bird species (Larson et al., 2015).

It is important to recognize that fear impacts human attitudes and behaviors toward keystone species, particularly those regarded as dangerous or harmful (e.g., wolves, bears, and big cats). Fear may limit the involvement of local communities in managing predator populations because of the high costs implied or because the social acceptance of certain techniques, such as reintroduction, may be difficult (Johansson et al., 2012). Examples of this include reintroducing wolves in Mexico and the United States, where emotions have played fundamental roles in the acceptance of new wolf populations (Straka et al., 2019). Mexican wolves (*Canis lupus baileyi*) were eradicated from the Mexican territory in the 1960s because of conflicts with farmers and negative perceptions due to livestock predation (Leopold, 1959; Moctezuma et al., 2004). Wolf reintroduction projects have been started recently in Northwestern Mexico, where it has been clear that social acceptance is the primary limiting factor for their success (Araiza et al., 2012; García, 2014; Lara-Díaz et al., 2015).

Society’s emotions toward wildlife may be key elements for decision-making on conservation issues. Anger is one of the primary collective emotions that can lead to positive changes for natural resource management when social pressure is put on government leaders to improve and enforce environmental legislation. However, anger may have other implications and cause social fragmentation (Buijs and Lawrence, 2013). In these cases, participation of wildlife management agencies is crucial given their social confidence. If the capacity of these agencies is not appropriate, collective distrust and fear of dangerous and disgusting animals may stimulate hostile environments for their proper management (Johansson et al., 2012). Community confidence in environmental agencies is especially relevant where threatened species are under recovery, as is the case with wolves in different countries (Swenson and Andrén, 2005), or where people take action by themselves, such as in the case of the killings of Andean bears (*Tremarctos ornatus*; Figueroa, 2015).

It seems clear that some wildlife species are far more significant to humans than others (Herzog and Burghardt, 1988), perhaps linked to their evolutionary closeness (e.g., primates, and particularly the great apes; Gunnthorsdottir, 2001; Miralles et al., 2019) or because of their cultural, aesthetic, or affective attributes favoring more interest and attention toward them. Interest and attention favor people’s attitudes for conserving these species, differently from others without a transcendental meaning for social groups. This idea highlights the relevance of designing conservation strategies fomenting interest for wildlife through generating affective links between humans and animals both in rural and urban areas.

Beautiful and attractive animals causing “positive” emotions (e.g., happiness and surprise) receive special attention driving *in situ* and *ex situ* conservation actions (Gunnthorsdottir, 2001). This could be a limitation for conservation efforts focused on species considered unattractive particularly in zoos. The preferences of human societies to watch specific animals have promoted that zoos keep attractive species more than those needing protection due to their conservation status (Frynta et al., 2010, 2013). Mammals constitute the preferred group among zoo visitors around the world (Moss and Esson, 2010). However, these spaces keep only 179,868 individuals belonging to 1,048 species (Frynta et al., 2013), which represent just 16.4% of known living species (Burgin et al., 2018). This preference is strongly biased toward large, attractive, and active mammals belonging to the families Ailuridae, Felidae, Phascolarctidae, Ursidae, Giraffidae, Elephantidae, Equidae, Macropodidae, Mephitidae, and Cervidae, among others (Frynta et al., 2013). The same correlation between human preference and species kept in zoos was found for large, colorful, and long-tailed parrot species (Frynta et al., 2010). In contrast, small and unpopular species do not motivate the same appreciation, even if they are endangered. As a consequence, zoos generally keep a few of those local species (Frynta et al., 2013). In this sense, zoos and other places keeping wildlife need to implement exhibition strategies to promote human interest on less attractive but highly relevant animal species of threatened ecosystems (Bitgood and Patterson, 1987; Frynta et al., 2009).

Considering this distinction in preference, it is relevant to spread information about the ecological importance of animals in ecosystems, especially regarding native and endangered species (Conde et al., 2011), Messages to promote “positive” emotions in people could be a way to support the appropriation of endangered species by societies and improve their attitudes toward them in the long term. Massive media communication may be of utmost importance for these purposes, especially if the appropriate images of and messages about target species are transmitted to the general public (Gunnthorsdottir, 2001). Following Breed and Moore (2016), successful conservation projects require focusing on promoting wide social empathy for wildlife species, particularly those that generate fear and disgust (e.g., large predators, venomous species, and many amphibians) motivating their killing or removal (Bishop et al., 2012; Prokop and Fančovičová, 2012; Prokop et al., 2016).

## Final Remarks

Individual and collective idiosyncrasies have promoted a diversity of attitudes toward wildlife species (Herzog and Burghardt, 1988) motivated in part by a diversification of emotions built with dynamic biological and cultural elements. Identifying and understanding diversified emotions and their local precursors (e.g., in areas where protected areas and human presence are relevant) would allow analyzing wildlife problems and their solutions through multidisciplinary strategies.

Considering that knowledge is a relevant element for the expression of emotions, we propose that regional strategies to integrate information on the biology, ecology, and management of culturally important animal species (particularly those regarded as fearsome, dangerous, harmful, and disgusting) should be included in national education systems and massive media campaigns throughout the Neotropics (Espinosa and Jacobson, 2012). These strategies must be carefully designed by taking into account the impact of mass media (e.g., news, television shows, documentaries, films, and public text books, among others) may have on the public about wildlife conservation (Røskaft et al., 2003; Knight, 2008; Ceríaco et al., 2011; Wieczorek, 2012). When an animal species is projected as aggressive, a negative emotional experience can be produced in the public. This negative experience may in turn lead the individual to believe the species is a dangerous agent or threat to human life, bringing about attitudes against its conservation (Prokop and Fančovičová, 2017). On the contrary, if wildlife species are positively seen by children through different media outlets, where the real facts about unpopular animals are shown, it is more likely that fear and disgust decrease, while empathy may grow (Prokop et al., 2011). Ensuring the continuity of transmitting traditional ecological knowledge about animal species will be equally important to stimulate positive emotions and a long-term interest of the new generations in wildlife conservation (Jacques-Coper et al., 2019).

Another strategy that could have a positive impact on emotions toward fearsome and disgusting animals is promoting physical interactions with them (e.g., touching snails, rays, amphibians, mice; Randler et al., 2012; Prokop and Fančovičová, 2016); the new knowledge about the animals and physical contact with them could reduce the anxiety of danger. Recognizing that emotions are culturally influenced, we propose developing outreach strategies by retrieving traditional aspects that formerly favored empathy with animal species, including the non-charismatic or unpopular ones, even if they are threatened.

This review aimed to discuss the role of emotions in the conservation of species which a have been transcendent for the human species throughout history and that in many cases are currently threatened by extinction. In particular, we stress that the social component is of utmost importance in wildlife conservation across Latin America, especially in megadiverse countries where ethnozoological studies have documented the relevance of human–wildlife relationships (Jácome-Negrete et al., 2013; Sarukhán and Dirzo, 2013; Manzano-García and Martínez, 2017).

## Author Contributions

NC-H wrote and edited the manuscript. EN translated and edited the manuscript. DS-F and EE-L edited the manuscript. All authors read and approved the final manuscript.

## Conflict of Interest

The authors declare that the research was conducted in the absence of any commercial or financial relationships that could be construed as a potential conflict of interest.
